# A collective narrative of care and complex mental illness

**DOI:** 10.1177/13634593251358048

**Published:** 2025-07-28

**Authors:** Tanya Park, Eduan Breedt, Megan Sommerfeld, Lindsay Komar, Nicole Tailby, Karlee Podritske, Tim Barlott

**Affiliations:** James Cook University, Australia; University of Alberta, Canada; University of Alberta, Canada; University of Alberta, Canada; University of Alberta, Canada; University of Alberta, Canada; University of Alberta, Canada

**Keywords:** care, caring, collective narrative, epistemic injustice, mental illness

## Abstract

Caring is a fundamental concept in healthcare, yet it is fraught with challenges for people living with complex mental illnesses (CMI). Many scholars theorize relationality and interdependence in their definitions of care, however, there has paradoxically been a sustained failure to involve the testimonies and voices of the people who clinicians are connected to and have interdependent relationships with. Using the work of feminist ethics of care scholars Berenice Fisher and Joan Tronto, we conceptualize care work through relationality and sensitivity to (in)justice. While foregrounding relationality and justice, we used a collective narrative methodology and collective documentation to create stories of care. By documenting stories of care, we hope to contribute to the conversations on care and caring practices.

## Introduction

Caring is a fundamental concept in healthcare, deeply ingrained in the thoughts and actions of nurses, and in the minds of people seeking healthcare. However, caring within modern health systems is fraught with challenges. Care that focuses on fixing rather than cultivating a relational response between people whose afflictions are shaped by socio-political forces and systemic inequities turn patients into objects to which tasks, technologies, and/or skills are applied. With the modernization of medicine, its ever-increasing demands, and fast-pace, care is rendered a system of checks and balances, a resource divvied out to those who are suffering. Economic austerity principles applied to healthcare delivery promote efficiency and devalue therapeutic and interpersonal connections (Pascal and Sagan, 2017). The increased commodification of care coerces nurses to distance themselves from their caring role in order to meet the demands of the workplace (Aulenbacher et al., 2018; McKeown et al., 2019). As Tronto states, “For much of the modern era, we have cared more about wealth and its production than about caring for people” (Tronto, 2020: 96).

The socio-political context of healthcare systems affects how care is enacted, what forms of care are valued (or devalued) in healthcare, and who or what is cared for. Care, as we will argue, can be good, but it can also oppress. Our project grapples with understanding how care flows for people living with complex mental illness, a group that is excluded and neglected in society. Using a collective narrative approach (Denborough, 2008), we document stories of care experienced by people with CMI.

### Care in healthcare

Care in healthcare has historically been understood and dichotomized into something given by the clinician and received by the client, reducing care to a resource that is either handed out or withheld by healthcare providers and systems (Nordhaug and Nortvedt, 2011; van Nistelrooij and Leget, 2017). For instance, care has been theorized as being a central practice in nursing, which centers the “caring” activities of nursing as a way of “being-doing-knowing” (Thompson, 2020: 49). This produces a binary and hierarchy between caregiver and care-receiver, naturally presupposing that the clinician has the power to give or withhold care. This dominant, hierarchical, and transactional paradigm of care in healthcare has been extensively scrutinized by feminist theorists and care ethicists (Griscti et al., 2016, 2017; Thompson, 2020). Yet contemporary healthcare scholars who draw from feminist ethics of care, continue to reinforce clinician-client binaries and power hierarchies when defining and understanding care.

### Living with a complex mental illness

In any given year, one out of five Canadians is likely to encounter a mental health issue (Canadian Mental Health Association, 2020). Some will experience complex mental illnesses (CMI). This term typically refers to disorders within the schizophrenia spectrum disorders (e.g. schizophrenia, schizoaffective disorder), bipolar disorders, and persistent depressive disorder. People living with CMI are disproportionately affected by social and structural barriers to care, such as poverty, substandard housing (Hwang et al., 2011; Jaworsky et al., 2016; Kim et al., 2007), unemployment (Rotenberg et al., 2022; Wicks et al., 2005; Zammit et al., 2010), undertreatment (McNamara et al., 2018), life-threatening treatment side effects (Correll et al., 2018; Hayes et al., 2017; Lambert and Newcomer, 2009; Mazereel et al., 2020), exposure to adverse and stressful life events (Matheson et al., 2013), and social disadvantage in childhood (Varchmin et al., 2021). People with CMI are frequently subject to profound social exclusion (Barlott and Setchell, 2022) and a decreased life expectancy of up to 25 years (Barican et al., 2022; Hennekens et al., 2005; Robson and Gray, 2007).

There exists a plethora of literature documenting and describing the challenges of treatment for people living with schizophrenia (Cohn et al., 2004; DeHart et al., 2006; Goff et al., 2005; Hasson-Ohayon and Lysaker, 2021; McEvoy et al., 2005; Stroup et al., 2003), and there is also literature describing the lived experiences of people with CMI (Kaite et al., 2015; Stenhouse, 2014). There is also the recent popularity in participatory methods that involve and directly collaborate with people who have mental illnesses in the research process (Calabria, 2016; Calabria and Bailey, 2023; Davidson et al., 1997; Torrissen and Stickley, 2018). In participatory research the stories of people with CMI are not controlled by researchers but they have control over authoring their own stories about living with CMI (Clubhouse of St. Joseph County, 2022; Ellis et al., 2021; Schneider, 2011). These participatory methods aim to subvert rendering the mental health patient passive and helpless often encouraged by biomedical research, making them active agents in their stories. To our knowledge, however, there is no documented collective narrative, as described by Denborough (2008), of care and caring with people with CMI.

## Developing our conceptual framework of care

Our perspective on care is guided by our experience as healthcare scholars and providers and the work of feminist scholars Fisher and Tronto (1990: 40), who conceptualize care as a “species activity that includes everything that we do to maintain, continue, and repair our ‘world’ so that we can live in it as well as possible. That world includes our bodies, ourselves and our environment, all of which we seek to interweave in a complex, life-sustaining web.” From Tronto’s definition we understand that care is a process, a verb, or a range of doings, that flows throughout our world, creating, sustaining and holding together life.

Caring, according to Maria Puig de la Bellacasa, “passes within, across, throughout things.” (de La Bellacasa, 2017: 1). For care to flow, care presumes that all of life, including human beings and things, do not exist outside of their relations with one another—rather they form a “complex, life-sustaining web” (Tronto, 2013: 103). Human beings are not self-contained beings that move through the world unaffected by the world, rather they are interdependent on the world and in constant flux with the world. For instance, noticing rain on your skin might cause you to slow down or speed up, changing the trajectory of your day. Care flows through collective sharing of perspectives and stories, a kind of joint action that continuously shifts the common epistemic ground beneath us, shaping our social world. A human’s existence is a result of their never-ending interdependent relations and interactions with the world.

A feminist ethic of care makes clear that we cannot care without understanding that the human is already a collective held together with care. We argue that caring requires a move beyond individualistic and potentially isolating ideas of care to a collective notion of care (Shevellar, 2015). Could this move change how we think about how healthcare is given and received?

## Collective narrative approach

Collective narrative is a practice as well as a research method that creates a context for individuals and communities who have experienced social suffering, and stigmatization to make contributions to the lives of others who are going through similar difficulties (Denborough et al., 2006; Shevellar, 2015). In many narrative approaches, such as narrative analysis (Riessman, 2008), a distinction is made between stories and narratives. Stories are specific tales people tell while narratives are social and cultural templates or “resources from which people construct their personal stories and understand the stories they hear” (Smith and Monforte, 2020). As we explore further below, Denborough’s (2008) collective narrative approach offers participants a “double storied” narrative with which people can use to construct and make sense of their, and other’s stories. While narrative methodological approaches, such as “narrative portraits,” centers the narrated and embodies experiences of individual people (Rodríguez-Dorans, 2023; Rodríguez-Dorans and Jacobs, 2020), a collective narrative approach centers a collective or community and the impact on community knowledge on stories of harm and resilience. Through a collective narrative approach, then, communities can contribute to the common ground and collectively accumulate knowledge that “puts voice to” and helps “make sense of” their lived experiences; “Telling our stories in ways that make us stronger” (Wingard and Lester, 2001).

Narrative, in collective narrative practice, refers to the centrality of people’s stories and the impact made through telling and retelling of stories (Denborough, 2008). Narrative approaches involve understanding the stories of people’s lives and collaboratively reauthoring these narratives through collective documentation (Denborough, 2008). The researcher in a collective narrative approach is not one of “story analyst” or “storyteller” (Bochner and Riggs, 2014; Smith and Sparkes, 2006) but of a facilitator and collaborator; stepping aside and making the space for people with lived experience to contribute their stories and knowledge to the common epistemic ground and collaboratively make sense of their stories. This reauthoring is a form of presenting stories in a manner that decenters the voice of the academic author and prevents the silencing of participants’ voices (Stenhouse, 2014). This approach allows an opportunity to collectively make sense of and share human experiences, and the complexity within our experiences, in the case of our project the experience of care and caring. This paper explores, acknowledges, documents, and shares the collective narratives of a group of people living with CMI.

### Collective narrative as an approach that centers epistemic justice

We used a collective narrative approach to document stories of caring, to make space for testimonial justice by giving name to and making sense of problems faced by people with CMI. The erasure of stories and knowledges is what critical philosopher Miranda Fricker called epistemic injustice or being “wronged” as a knower—where someone is not believed or their knowledge is rendered invalid (Fricker, 2010; Johnstone, 2021). Often, societal processes dictated by powerful institutions (such as psychiatry, research institutions, industrial medical complex, and societal stigma), exercise power over mental health discourses through the management of knowledge, determining whose knowledge and stories get told, how they get told, and whether they are deemed valid or invalid (Johnstone, 2021). AsDenborough (2018: 8) says, “The stories we tell about ourselves are not created in a vacuum. All too often, the stories we believe about ourselves have been written by others.” Using a feminist ethics of care, we argue that care work is constituted by relationality and sensitivity to injustice where people with CMI collectively address the injustice of having their voices silenced.

Collective narrative approaches attend to damage-centered injustice using, what Denborough terms, “double-storied testimony,” a process where one simultaneously listens to a single narrative for two distinct aspects. When we focus on one facet of a narrative or testimony, it often unveils accounts of societal suffering, including the enduring effects and injustices stemming from such suffering, as well as the formidable obstacles that perpetuate this suffering. Conversely, when we shift our attention to the “second” story within the narrative, it unveils stories of response and resilience. This double-listening approach not only highlights the individual’s acts of resistance but also unveils their endeavors to protect, nurture, and assist others (Denborough, 2008). Through a double storied approach, we not only document the painful aspects of social reality, but “also the wisdom and hope,” representing people and communities as more than “broken and conquered” (Tuck, 2009: 416). When individuals with CMI share their stories and lived experiences, they disrupt and refute the dominant societal assumptions about them. Through collective sharing, telling and retelling of stories, information, and knowledge is added to the collective common ground, reshaping our social terrain and ways of acting. Epistemic justice, then, is not always about “discovering” something new but a deliberate act that ensures that voices that are systemically erased are uplifted and included. A collective narrative approach understands that “we collectively build the world together” when we successfully challenge the common ground with words and deeds and as a result, we change the social environment itself (Táíwò, 2022: 60).

### Research aim

This project used the collective narrative approach to answer the research question, “How is care experienced and understood for people with complex mental illness?”

## Method

Collective narratives were developed in two narrative triads (groups of three). Each member of each triad (including research team members) had lived experience with CMI. The groups were people who all had a self-identified complex mental illness. One person in each group of three was also a member of the research team who also experiences CMI.

We used a purposive sampling approach to recruit participants. The study was advertised through personnel networks, and digital posters on social media. Interested people were invited to contact the research team for additional information. Ethics approval was received from the University of Alberta Human Research Ethics Board.

### Collective narrative development (data collection and analysis)

**Figure 1.**
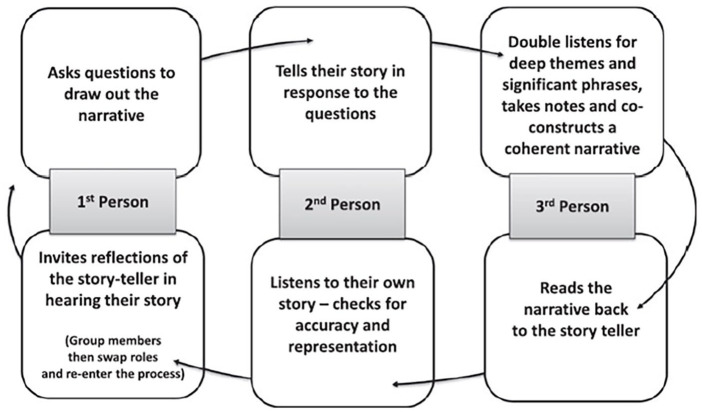
Collective narrative process (Shevellar, 2015).

The data collection procedure followed a cyclic process that people in the triad moved through from first person to second person to third person within the process. The process of collective narrative is outlined in Figure 1. Collective Narrative Process.

There were three group meetings to develop, review and discuss the collective narratives. We developed a process to set up the meetings in a democratic way, intentionally inviting a research team member from outside of the triad to introduce the process at the beginning of each meeting and then leaving. This was to ensure that the focus group shared power and remained as non-hierarchical as possible.

The set of questions that were used as prompts in focus group meeting 1 were, guided by four themes, taken from Denborough (2008). The four themes are, starting with a “skill,” locating the skill within a story, embedding the story in history, and then linking it to the collective. Denborough (2008: 29) argues that these four themes facilitate stories to be “more richly collective” and “more clearly linked to cultural contexts.” Based on these four themes, the following questions guided the collective narrative conversations:

1. What is care to you? What is one thing you value of care, caring, or being cared for?
2. Can you share a story about a time you experienced care or caring that made a difference to you or others?
3. Where did you learn about care and caring? Who did you learn it from?
4. Who in your life would support your decision to work on standing up for your own / or other’s care?
5. Is your experience of care/caring linked with any community or cultural traditions in your life/community/family/culture?
6. How has society affected your ability to care or be cared for?
7. What does your care story say or not say about professional care given or received?

It was emphasized to the participants that there is no “correct” way to conduct or participate in the narrative group, but rather that the group is flexible and should be guided by the dialog that emerges. Participants were reassured that the questions were prompts that may be reiterated or modified, and that participants may choose to not answer or skip questions if they prefer.

### Developing the collective narrative

Narrative group meetings were recorded with permission and transcribed. The collective narratives were organized by the researchers involved in the triad focusing on drawing out double-storied testimony, highlighting both suffering and resilience in the face of suffering. Common themes, patterns, and variations were grouped together, looking for similar double stories of harm, and the skills learned from these experiences to produce a relatively cohesive collective narrative document. The initial draft of the collective narrative document was compiled by the research team member of the triad. Following Denborough (2008), the collective narrative intentionally avoids flattening differences between experiences by using “I” or “one of us” to signify an individual’s unique story, and “we” when an experience was shared. Once a draft collective narrative was created it was shared with the participants involved and two subsequent meeting were organized in order to revise the written collective narrative. In these meetings, triad members read aloud the narrative and shared their reactions and thoughts. The triad members were invited and encouraged to make any changes or additions to the document to ensure it was accurate and that their individual and collective voices and stories were heard in the text. All triad members agreed they could. The final document includes the experience and stories of all. The collective narratives are presented using headings and quotes for emphasis. The collective narratives presented here are the stories of people with lived experience of CMI.

## Collective narratives of care

### Learning how to feel connected

Many of us have been harmed by the medical system: **“**I have had some more negative experiences with care teams that have led to trauma in terms of my relationship with the healthcare system. There have been instances where I have not felt the most cared for and that has ultimately impacted my ability or desire to reach out for support from the healthcare system. And so yeah it is definitely important, you know relationships and interactions that you have with health care professionals in terms of influencing or informing how you continue to engage with the care system going forward.”

To overcome trauma, we have learned that making connections and building relationships will help to mitigate these situations. One of us stated “The nurses, the doctors, the psychiatrists they help me to live a better life. Even though now I don’t have encounters with them they are still part of my life because even now with this study they are helping me to receive extra money and helping me to see how others are living with a similar disease that I have.” Some of us learnt that although the system is harmful there are professionals who can help us. “When I was being cared for by a psychiatrist [at an] inpatient hospital she really did a great job of meeting my needs in terms of, you know, really trying to understand where I was at, and trying to work with me from where I was at, and she took a really great approach that felt good to me of being really honest with me and working with me towards the goals that I was communicating that I had.”

Connection is not only needed when in the healthcare system. We as human beings value connection above all else. The need to feel close to someone. The need to talk to someone. The need to not feel alone. One of us stated “I live in a pretty good community. I get pretty good support from neighbors and different communities I [have] lived in that help with my care [by] socializing with me.” Another one of us learned to make connections while in university. “When I was in University, I had professors and school staff, who really did a good job of caring for me [by] trying to understand how I was feeling in any given situation, and kind of sit with me in that and kind of validate my experience, and then listening to what my goals were and what I wanted to move forward.”

Another way we learned to build connections was through reciprocal care. One of us vocalized that the experience of reciprocal caring and being *shared with* allowed them to feel cared for. For one of us, it was integral to the development of a caring relationship with a nurse while in the hospital. They go on to say, “I was involuntarily inpatient, and this happened to be right after, I was really in the throes of psychosis and I got word that my dog died while I was in the hospital, and I just, I completely lost any semblance of control.” One of us vocalized that in her time of vulnerability, one of her nurses saw this vulnerability and made an intentional effort to make them feel cared for. “One of the nurses came, she spent just a lot of time sitting with me, sharing some of her own stories, I didn’t feel like I was being talked to, I was being shared with and that really made me feel cared for and understood.”

### Finding and making ways to feel safe

Stigma toward mental illness exists in society and impacts our experiences with the healthcare system on a daily basis. These experiences lead to us not feeling heard, which leads to trauma with the system. This trauma impacts our ability and desire to reach out for support when we need it. “I think that something that I face as someone who’s living with a mental illness like schizophrenia/schizoaffective for me has been kind of the stigma that exists within society, and how that has kind of impacted my ability to be cared for in some circumstances where it felt like I maybe couldn’t ask for help or I couldn’t accept help because of that stigma that exists. It makes it more difficult and then you know, obviously other times there was stigma involved where people didn’t know how to care for me.” One of us vocalized the impact stigma had on our understanding of illness by referring to symptoms of the illness as a normal part of growth of development. One of us stated, “the initial response was oh, she’s just questioning her sexuality” when describing her parents’ thoughts when she first started describing her intrusive thoughts.

One of us spoke about the impact of societal stigma and how this has had an impact on our perceptions of ourselves and our ability to receive care. “In the context of living with an illness like schizoaffective disorder, there is a lot of stigma within society about what it means to live with that and I think that has kinda gotten in the way in more of a negative way, in terms of impeding my ability to be cared for or even accept care because I think there is some of that internalized stigma too that has kept me from feeling like I deserve care or feeling like I can accept it in an effective or meaningful way.” One of us goes on to describe an experience of creating a supportive community for themselves and developing a greater understanding of reciprocal care. One of us argues that from our hardship, we were able to become selective in whom we participate in caring relationships with, stating, “I guess I have created my own kind of supportive community that has really helped me to understand the caring dynamic and the caring relationship, and I am able to accept care from those close people in my life, but I’ve also learned how to kind of reciprocate that as well and offer care as well and learn more about the reciprocal dynamic of caring relationships.”

One of us vocalized the experience stigma has had on their family’s understanding of their illness, stating “my mother, she doesn’t, I don’t think, fully understand my diagnosis and so just keeps saying, well um well, you’re better now right and like everything should just go away like I got over a cold.” One of us reports that in response to their family’s lack of understanding, they seek out support elsewhere, in this case from their ex-husband. “Whereas my ex-husband, he has been a really big advocate for me, and I can rely on him if I need to call somebody and talk about things because he understands.” One of us reports that due to their mother’s lack of understanding, their mother is not able to give care to the same extent as her ex-husband, who has a better understanding of their lived experience “he has seen me at my worst and gone through a lot of really difficult things with me.”

As a result of stigma, we have learned how to find safety and comfort, even while hospitalized, by finding other people with similar problems. “In general, it felt good because I felt safe in the institution. I could sleep better. I could eat better. I didn’t feel alone because there were others who had similar problems that I had.” We are also comforted in knowing that others value our safety. “Especially during the rough times, I learned that my family would care for me and that they valued my life and my safety.” And care from professionals too: “they valued what I was going through, if I had eaten, if I was feeling well, if I was safe or not safe.”

### Access and advocacy

We all have experienced problems with navigating the system and trying to get access to resources that we need. “I’ve had a rough time finding proper support from professionals where I live. Well, it’s been a battle with that, finding proper support.” These hardships have taught us the importance of finding advocates to help you through the process. One of us then spoke of the need to learn to advocate and educate our caregivers in order to receive care, saying, “really advocating for myself, like no this is actually a manifestation of my [CPMI].” In return, one of us vocalized that in working through our caregiver’s stigma and misinformation allows us to feel cared for “My parents being willing to listen and hear me out where I was at really made me feel cared for.” One of us stated “My sister, my dad, my mom, and my wife were really behind me. They supported me and fighting for things that were available for me in society that could help me live a better life.” Another one of us stated “My husband has definitely done a really good job of caring for me in this sense, helping me, advocating for me and helping me advocate for myself in accessing care.”

These supports mean everything to us because without them we could not survive. “It affected me positively because I received money from the government, and without it I wouldn’t be able to survive because I cannot work full time and I help part of my family that needs money as well to survive. I guess they could improve certain things, but overall, they’re helping me.” And another one of us states “then I found my therapist, who I adore, she has been so foundational in like me being able to function and accept how I’m living my life and accept all my intrusive thoughts, she’s been foundational” and then “I graduated [OCD specific] therapy, but she’s still like, I’ll be your therapist for life, she’s still willing to help me with things that aren’t specific to my OCD which I really appreciated, so it wasn’t like I only work with this one part of you, it’s like I want to see the whole person which I really appreciated.”

### Chosen family

Some of us grew up in nurturing homes that fostered authentic communication and unconditional acceptance among family members. We learn that this is not a universal experience; not everyone receives the nurturing care they need from their biological family. “I don’t necessarily come from a family that shows their feelings very well or even is very open with feelings and with relationships. . .like, I have a nut allergy and it’s something that was kind of ignored growing up or like not taken seriously. It might be my birthday and my mom will be busy so she’ll go to Costco and for dessert just bring back a bunch of squares that I actually can’t eat. Even this morning I showed up at the lake and she’s like “here, eat these cherries,” and I’m like, “Mom I’m allergic.” Sometimes this lack of nurturing can influence how we perceive care and prevent us from accepting care from others: “My family definitely cares about each other and loves each other, but they don’t necessarily care *for* one another and so I struggle with accepting that from people even to this day.”

Growing up in a care deficient environment does not mean we are unable to care for ourselves or others. We find our way to care using alternative paths. The love and support we need can be found or chosen among the people we are bonded to: “For me, my life is very much like my chosen family [. . .] I think that Community is very very important. If you can find it and if you can foster it and if you don’t it’s extremely difficult, if not impossible to care for somebody else if you don’t have a community that can also care for you or can help you care for that other person.”

Finding the care we need outside of our biological families can help teach us how to care for the people we love, “I think that the act of being able to care for others or understanding how to allow others to care for me is something that I learned as an adolescent and as a teenager from other models outside of the one I grew up in.” Most importantly, it also teaches us how to care for ourselves, “I realized that my family never talks about things. . .maybe that’s why it’s so important to me now, to be heard. Being heard and being able to hear people [is] just about being accepted for who you are. . . those are the experiences that leave me feeling most nourished.”

## Discussion/conclusion

The collective narratives are guided by intentional conversations about care and caring. They invite us to reflect, as healthcare providers (both with and without experiences of mental illness) about the ways we could better offer care or support our patients in receiving care. Below, we put the collective narratives in conversation with the literature.

### Learning how to feel connected

We notice that this narrative theme mentions “negative experiences with care teams that have led to trauma,” yet the participants chose to focus their narrative on their positive experiences with psychiatric services. While we could consider this a form of desirability bias and presenting a story that they think is favorable to the research team, we also recognize generosity and grace in these stories.

We receive this collective narrative as an invitation to connect and be a part of their interrelated network of connections. When healthcare providers connect with patients “where they’re at” it can have a healing impact. In fact, it is well documented that the primary and most consistent driver of positive outcomes in mental health interventions is therapeutic relationship (Cahill et al., 2013). Healthcare providers are a part of patients’ “life-sustaining web” (Fisher and Tronto, 1990), especially since we are often involved when things are most in crisis. Still, we do not want to ignore the other aspects of this narrative. Yes, healthcare providers can play a role, but this narrative articulates a wide network of community. Participants spoke of people like neighbors, teachers and professors that supported them to “move forward” (Eppler, 2024).

The narrative shares an example of an in-patient experience, where a participant’s dog died, and a nurse took the time to sit and shared stories together. Both parties embodied a feminist ethic of care, recognizing that all life, and our experiences in the world, are held together by care. By collectively sharing stories, they made sense of, shaped, and were repairing their world (Fisher and Tronto, 1990; Frank, 1997). There is a common perception in healthcare that there isn’t time to care in this way (Aulenbacher et al., 2018; Tronto, 2020). But, we wonder, are we complicit when we choose to use our time in the ways neoliberal health systems expect us? Was this nurse reprimanded for taking the time to care? We do not want to individualize or put the burden on healthcare providers to combat neoliberal service logics, but we do wonder if providers could be more attentive to affirming the humanity of their patients (Eppler, 2024). Reciprocity does not necessarily mean increasing the “workload” of the provider but invites questions on how we are compelled to think of care in the midst of our existing workload. *Is the dynamic of caregiver and care-receiver linear, static, binary, or mutually exclusive? Or are both positions simultaneous and always being negotiated? How is this lived and felt through the stories we tell/invite with our patients as we go about our tasks? How often do we pop our heads into their room on our way home to say, “see you tomorrow” and drop off an extra warm blanket?* The narratives illustrate how caring as a reciprocating act enables dynamic caring actions to take place, creating a community of carers, and an opportunity to both offer care and accept care.

### Finding and making ways to feel safe

This narrative theme is mainly about stigma but framed with a heading about feeling “safe.” The loss of reciprocity between the patient and the clinician often leads to stigma, and disempowerment (Hutchinson and Lovell, 2013). With mental health stigma and discrimination people experience a downward spiral of structural barriers and worsening health (Alegría et al., 2018; Livingston, 2013). This narrative is an invitation to consider the modes that stigma plays out in our services. While care can be a positive force that forms new relations, care has also been stigmatizing, violent, and destructive. A feminist ethic of care has revealed “how relations of care can be cruel as much as loving, unpacking what is actually done in different situations under the blanket category of care” (de La Bellacasa, 2017: 11). While the very best intended care worker may seek to provide care for people with CMI, the very act of being a nurse may render the care worker complicit in systems that perpetuate exclusion and discrimination. Merely prioritizing risk management protocol over the patient, for instance, can quickly disrupt therapeutic relationships and begin a cycle of discrimination (Felton et al., 2018). Also, consider how being “evidence-based” in one’s approach implies that one’s knowledge is drawn from resources deemed valid and reliable. If that source of knowledge does not include the perspectives and voices of people with CMI, which it often doesn’t (Russo, 2023), one’s care work practice reinforces the dominant status quo, risking marginalizing, and stigmatizing those with CMI. Therefore, justice-oriented caring always involves carefulness with stories, testimonies, voices, and knowledge.

Furthermore, mental health practices often center (and manage) behaviors rather than recognizing that behaviors are rooted in distress, trauma, and often serve a purpose (Burstow, 2003, 2005). The narrative notes the role of internalized stigma, and that this plays a part in whether people can receive just care. Therefore, we must not romanticize care or consider care as a relational force as good in and of itself. To be *cared for* and *to care* can be generative and affirming, and it can also be oppressive (de La Bellacasa, 2017: 1). Paying attention to power, oppression, and injustice is essential to care (Laurin and Martin, 2022: 3; Tronto, 2013: 140).

It is important to note that care sometimes involves the rejection of a system or institution that is harmful. That is, where harm is enacted in the very places where care is expected to exist and radical acts of caring sometimes involve cutting off relations to enable care to flow once more in new ways. Many people with lived experience of CMI already know this; they have experienced oppressive care within institutions (Russo and Beresford, 2015). Therefore, justice-oriented care always has a dimension of refusing care and refusing to care when one is compelled to. There is a disruptive power in refusing care or choosing not to care about what we are forced to (de La Bellacasa, 2017: 5). This refusal is what de La Bellacasa (2017) calls a “critical cut into a thing,” a detachment of a part of a caring relation so that different relations can be constructed, “critical cuts don’t merely expose or produce conflict, they also foster caring relations” (de La Bellacasa, 2017: 62).

This narrative theme ends with what we consider to be an invitation to care. Whether or not patient’s seem recipient to care (or refuse), we have a responsibility to their safety when in our care, to see if they have enough to eat, to see if they are sleeping ok, and maybe even asking “do you feel safe here?”.

## Access and advocacy and chosen family

This narrative theme tells a story of participants surviving with the allyship of people who are invested in their life, and in turn people they are invested in. Despite the narrative primarily highlighting the ways that others support and care for them, access and advocacy occurs within mutual and committed relationships. Their survival is necessitated by mutuality of relationships (Alber and Drotbohm, 2015; Levin et al., 2020). The term “chosen family” originated from the work of Kath Weston’s *Families We Choose: Lesbians, Gays, Kinship* and is most prominently deployed in queer and transgender communities. Chosen family is a radical alternative means of forming a supportive community that subverts the bio-legal classification of family (Levin et al., 2020). For some who continue to experience medical trauma and discrimination, kinship and the support and advocacy of chosen family is crucial to enable them to continue attending lifesaving medical care (Levin et al., 2020). Kinship is a distinct and radical form of care (Alber and Drotbohm, 2015). In the collective narrative, care is received and learned from mutuality, networks of care, and chosen family, where they are “heard” and in turn “hear.” The narrative points clearly to the mutuality of care. This speaks to the value of intentional community, mutual aid, and that care most often (and most genuinely) is received and given in mutual relationships with others.

While care is a central practice for nurses, it exists beyond nursing and healthcare institutions and permeates collectives and communities. Care is not exclusively a product of an institution or a profession but is entangled in the world, flowing in multiple directions between multiple actors at once. These collective narratives show us that the complexity of care outside of healthcare is necessary to keep people alive and to keep them living. Theorizing about care in the confines of an institution or a disciplinary field, to the exclusion of a segment of the population involved, neglects the richness of what caring does, misrepresents how care operates to hold the communities, people, and world together, and continues to marginalize voices that have historically been excluded from public discourse. So, while healthcare providers have some capacity for contributing to the care of people, it is in community (amidst family and kinship networks) that people with experiences of distress, trauma, and mental illness most receive and give care. We, as healthcare providers, can more intentionally integrate our patients’ care networks in our work (Eppler, 2024).

## A final reflection

Despite our best efforts to decenter ourselves as researchers and attend to relationality, justice, and the feminist ethics of care we acknowledge that subtle forms of silencing occur in our academic work, when we speak on behalf of others, mold knowledges and stories to fit the restrictive academic requirements of our academic occupation (Dimitrova, 2021; Russo, 2012, 2023; Stenhouse, 2014). Despite sharing responsibilities, sharing roles, and working democratically we cannot hide from the power of the academic institution and those who work there. Many scholars who discuss mental health and care do so while excluding people who have testimonies and narratives since they are deemed outside the acceptable bandwidth of intelligible perspectives (Russo, 2023). As a result, there is an ongoing failure to uphold the distinction between marginalized perspectives and those of allied scholars (Russo, 2023). As Russo (2023: 153) writes, “We often find ourselves giving not just our story but also the knowledge that has emerged from our experiences only to have it re-framed, serving various purposes and different agendas, and ultimately alienated from us.” In the case of this research, it must be noted that all authors are also healthcare providers who have a particular perspective and implicit academic agendas not shared by those who have CMI. Furthermore, while we tried to separate the researcher from their role in the triads, there seemed to remain an implicit expectation that the researcher leads the focus group thereby shaping the power dynamics in the group. At times this led to the researcher becoming very aware of power and their role, often impeding their ability to participate in a fulsome manner as a participant. However, once everyone in the triad began to share vulnerably about their experiences with CMI power hierarchies seemed to quickly flatten out, facilitating a democratic and spontaneous sharing circle.

The collective narrative in this paper has invited us, as healthcare providers, to deepen how we think about care. It is our belief that it would benefit all healthcare professionals to acknowledge the complexity of care, the harm institutions inflict on people with CMI, and collaboratively experiment with care that is both relational and just. Our hope and intention is that this paper is a step toward epistemic justice, adding the voices of those who experience CMI to the epistemic common ground. A feminist ethic of care is desiring of a more hospitable society, where when people experience distress, they “have a place to go where (they’re) loved” (Shamrat, 2013: 156), and, when they are in our care, that they experience *care.*
